# Direct and Rapid Detection and Quantification of *Oenococcus oeni* Cells in Wine by Cells-LAMP and Cells-qLAMP

**DOI:** 10.3389/fmicb.2018.01945

**Published:** 2018-08-17

**Authors:** Verónica Soares-Santos, Isabel Pardo, Sergi Ferrer

**Affiliations:** ^1^Estructura de Recerca Interdisciplinar en Biotecnologia i Biomedicina (ERI BIOTECMED), Universitat de València, València, Spain; ^2^ENOLAB, Universitat de València, València, Spain

**Keywords:** detection, quantification, *O. oeni*, grape must, wine, cells-LAMP, cells-qLAMP

## Abstract

Fast detection and enumeration of *Oenococcus oeni* in winemaking are necessary to determine whether malolactic fermentation (MLF) is likely to be performed or not and to decide if the use of a commercial starter is needed. In other wines, however, performing MLF can be detrimental for wine and should be avoided. The traditional identification and quantification of this bacteria using culture-dependent techniques in wine-related matrices require up to 14 days to yield results, which can be a very long time to perform possible enological operations. Loop-mediated isothermal amplification (LAMP) is a novel culture-independent technique that amplifies nucleic acid sequences under isothermal conditions with high specificity and efficiency in less than 1 h with inexpensive equipment. We designed LAMP primers for the specific detection and quantification of *O. oeni* cells. The developed LAMP method allows *O. oeni* to be detected directly from both grape musts and wines within 1 h from the time that the LAMP reaction begins, and without DNA extraction and purification requirements. The high sensitivity of LAMP methodology is achieved by previous mechanical cells lysis with no further purification by detecting one single cell per reaction in culture media, and in white/red grape musts and wines by avoiding reaction inhibition by ethanol, polyphenols, and other wine inhibitors. Cells can be concentrated prior to the LAMP reaction to further increase this sensitivity. Moreover, the LAMP method does not require expensive equipment and can be easily operated. The developed method is both economic and fast and offers high sensitivity and specificity.

## Introduction

In winemaking, *Oenococcus oeni* is the lactic acid bacteria (LAB) species most often involved in malolactic fermentation (MLF). This process consists in a biological wine decarboxylation process in which dicarboxylic L-malic acid is converted into monocarboxylic L-lactic acid and CO_2_. This deacidification reaction is usually required after alcoholic fermentation to produce most red wines and is desirable in some white and sparkling base wines because it improves their organoleptic properties and microbiology stability (Lonvaud-Funel, 1999; Liu, 2002; Wei et al., 2018). Fast detection and enumeration of *O. oeni* are necessary to determine whether MLF is likely to be performed or not and to decide if it is necessary to use a commercial starter or to even reinoculate. In other wines, however, performing MLF can be detrimental for wine and should be avoided in, for example, low-acidity wines, or when bacteria grow in bottles and muddy wine. The identification and quantification of these bacteria in wine-related matrices rely on traditional methods like culturing. Nevertheless, traditional methods require up to 14 days to yield results, which can be a very long time to perform possible enological operations. Attempts have been made to develop and use culture-independent techniques for detecting and quantifying *O. oeni* to, thus, avoid the problems associated with culture methods (Zapparoli et al., 1998; Rodas et al., 2003; Pinzani et al., 2004). Loop-mediated isothermal amplification (LAMP) is a novel culture-independent technique that was described by Notomi et al. (2000). It amplifies nucleic acid sequences under isothermal conditions with high specificity and efficiency in less than 1 h with inexpensive equipment (Notomi et al., 2000). The method requires a set of four specially designed primers that recognize six distinct regions of the target. This method relies on autocycling strand displacing DNA synthesis by *Bst* polymerase (also called *Gsp* polymerase). The large *Bst* polymerase fragment from *Geobacillus stearothermophilus* can amplify DNA by tearing off double-stranded DNA to yield a single strand. The combination of the DNA polymerase and the primer structure enables the amplification of target DNA at a steady temperature (between 60 and 65°C) (Notomi et al., 2000, 2015; Niessen, 2015). The final product in LAMP is a mixture of stem-loop DNA with various stem lengths and cauliflower-like structures and with multiple loops formed by annealing between the alternately inverted repeats of the target sequence in the same strand (Notomi et al., 2000; Parida et al., 2008; Tomita et al., 2008). Several detection methods that include dye fluorescence, gel electrophoresis, turbidity, and colorimetric change can be used to measure or otherwise detect target amplification (Parida et al., 2008; Kumar et al., 2017). Although many works are focused on the detection of the target microorganisms in food, more recently there is a great interest in using qLAMP as an enumeration or quantification technique, mainly for pathogens (Law et al., 2015; Kundapur and Nema, 2016; Garrido-Maestu et al., 2017; Hameed et al., 2018). Although some LAMP reaction can also be implemented even after eliminating DNA extraction and, thus, considerably cuts the overall assay time and reaction cost (Poon et al., 2006; Hill et al., 2008; Dugan et al., 2012). Nevertheless, wine is a complex matrix that contains DNA amplification inhibitors, such as ethanol, polysaccharides, pigments, and a wide range of polyphenolics (including tannins), which produce false-negatives (Wilson, 1997; Tessonnière et al., 2009). To date, the LAMP application from wine samples requires some form of nucleic acid extraction from the sample prior to starting the reaction (Hayashi et al., 2007).

The aims of this study were to design new primers and to adapt the LAMP methodology for specific, rapid, and easy *O. oeni* cells detection and quantification by directly sampling white and red grape musts, and wines, with no DNA extraction steps.

## Materials and Methods

### Strains and Growth Media

Different species of LAB, yeasts, and acetic acid bacteria (AAB) were used in this study (**Table 1**).

**Table 1:** The strains used in this study.

	Microorganisms	Strain
LAB	*Lactobacillus brevis*	CECT 216
	*Lactobacillus hilgardii*	CECT 4786
	*Lactobacillus plantarum*	CECT 748^T^
	*Leuconostoc mesenteroides*	CECT 394
	*Oenococcus oeni*	CECT 218
	*Pediococcus damnosus*	CECT 4692
	*Pediococcus pentosaceus*	CECT 4695
Yeasts	*Brettanomyces bruxellensis*	CECT 1451^T^
	*Saccharomyces cerevisiae*	ENOLAB 5022
AAB	*Acetobacter aceti*	CECT 298^T^
	*Gluconobacter oxydans*	CECT 4009

*LAB, lactic acid bacteria; AAB, acetic acid bacteria.*

*Oenococcus oeni* and AAB were grown in MLO (Caspritz and Radler, 1983), and *Lactobacillus* spp., *Leuconostoc mesenteroides*, and *Pediococcus* spp. were grown in MRS (Scharlau, Barcelona, Spain) supplemented with 0.5 g/L cysteine (Merck, Darmstadt, Germany). Yeasts were grown in YPD (Soares-Santos et al., 2018).

These strains were routinely grown in liquid medium at 28°C for 3–5 days. On a daily basis, the number of cells per mL was determined by microscopic counting in a Neubauer chamber until the population reached 10^8^ cells/mL.

### Grape Must and Wine Inoculation

White grape must (pH 3.20) and wine (10.22% ethanol, pH 3.20) of the Chardonnay grape variety, and red grape must (pH 3.27) and wine (9.86% ethanol, pH 3.23) of the Bobal grape variety, previously sterilized by filtration, were inoculated at 1% with the *O. oeni* species from the liquid medium in independent experiments.

### Cell Suspension Wash

All the *O. oeni* cell suspensions from the culture medium, grape musts, and wines were washed by centrifugation according to the protocol of Soares-Santos et al. (2017). Briefly, the cell suspensions obtained from the culture media were washed in Milli-U water (1 vol.), the cell suspensions from the white grape must and wine were washed in Milli-U water and 10% TEN buffer (0.1 M Tris-HCl pH 7.5, 0.05 M EDTA, 0.8 M NaCl) (1 vol.), and those from the red grape must and wine were washed in Milli-U water and 10% TEN buffer supplemented with polyvidone 25 (Merck, Darmstadt, Germany) (1 vol.). Cells were finally washed twice with milliU water (1 vol.). Whether convenient, cells can be concentrated 10× or 100× in the last centrifugation by resuspending in 0.1 or 0.01 vols.

### DNA Extraction

The genomic DNA from each species from the culture media, at a final concentration of 10^8^ cells/mL, was extracted with the commercial Ultra Clean^®^ Microbial DNA Isolation Kit (MO BIO, CA, United States) according to the manufacturer’s instructions.

### LAMP Primers Design

For specific *O. oeni* amplification, six primers were designed based on the 16S rRNA gene. The nucleic acid sequence of the 16S rRNA gene of *O. oeni* was searched in the GenBank database, and the accession number of the sequence was NR_040810.1. The sequence was further analyzed by the LAMP Designer 1.13 software to design the LAMP primers: two outer (F3 and B3), two inner (FIP and BIP), and two loop (loopF and loopB).

### LAMP Reaction Conditions

The LAMP reactions were performed using *Bst* polymerase 2.0 (New England BioLabs) following the conditions suggested by the manufacturer. The LAMP reactions were carried out in a total volume of 25 μL. Each reaction contained 1.4 mM of dNTPs, 0.2 μM of each outer primer, 1.6 μM of each inner primer, 0.8 μM of each loop primer, 8 mM of MgCl_2_, 1× Isothermal Amplification Buffer, 0.4 U/μL of *Bst* polymerase 2.0, and 2.5 μL of extracted DNA or 12.5 μL of cells (Cells-LAMP). The LAMP amplifications were carried out in a water bath, a heat block, or in a Mastercycler Personal 5332 (Eppendorf), which operated at a constant temperature of 62°C for 1 h. Negative controls were included at all times. The amplified products were analyzed visually by direct turbidity detection. To confirm the results, the amplified products were also resolved by agarose gel electrophoresis [2% (w/v) in 0.5× Tris-borate-EDTA buffer], stained with 0.5 μg/mL ethidium bromide and visualized in GelPrinter Plus (TDI) equipment. A 1 kb plus DNA Ladder (Invitrogen) was used as the size standard.

### Assessing LAMP Assay Sensitivity in Wine

The cell suspensions of *O. oeni*, at a final concentration from 10^2^ to 10^8^ cells/mL, were prepared in the white and red grape must and wine matrices. After washing the cell suspensions, each dilution was used as a template for the LAMP assay. Besides whole cells, the mechanically lysed cells were also assayed (Soares-Santos et al., 2018). For this purpose, after washing cells 425–600 μm-diameter glass beads (Sigma, St. Louis, United States) were added to each cell suspension [50% (w/v)], and each dilution was washed and lysed separately. Tubes were shaken in a horizontal microtube vortex-genie 2 (Scientific Industries, Bohemia, NY, United States) for 30 min at the maximum speed. The LAMP amplifications were performed with the designed primers in independent experiments using the whole or the mechanically lysed cells. Sensitivity tests were repeated twice.

### Cells-qLAMP Reaction Conditions

The Cells-qLAMP reactions were run in a total volume of 25 μL. Two different model systems were used for the comparison of the SYBR Green I and SYTO-9 fluorescent dyes. Each reaction contained 1.4 mM of dNTPs, 0.2 μM of each outer primer, 1.6 μM of each inner primer, 0.8 μM of each loop primer, 8 mM of MgCl_2_ (Invitrogen), 1× Isothermal Amplification Buffer, 0.4 U/μL of *Bst* polymerase 2.0 (New England BioLabs), 11.5 μL of the mechanically lysed cells, and 0.4 μM of SYTO-9 (Invitrogen) or 1 μL of 10× SYBR Green I (Invitrogen). The qLAMP amplifications were performed in triplicate in a C100^TM^ Thermal Cycler, CFX96^TM^ Real-Time System (BioRad), which operated at a constant temperature of 62°C for 1 h. Fluorescence signals were collected every minute, followed by a melting curve analysis obtained by slow heating from 60°C to 95°C at 0.5°C every 5 s, with continuous fluorescence collection. During the amplification, the fluorescence data were obtained in the six carboxyfluorescein (FAM) channel (excitation at 450–495 nm and detection at 510–527 nm). The RFU threshold value was used, and the threshold time (Tt) was calculated as the time at which fluorescence equaled the threshold value. The data analysis was carried out with the BioRadCFX Manager Software (version 2.1; BioRad). Negative controls were included at all times.

### Cells-qLAMP Standard Curves

Standard curves were created by plotting the Tt values of the Cells-qLAMP against different concentrations of cell suspensions (10^2^ to 10^8^ cells/mL). Efficiency (*E*) was calculated on the basis of the standard curve slope by equation *E* = 10^−1/slope^ − 1, as recommended by Bustin et al. (2009).

### Detection and Quantification of *O. oeni* Cells in Real Wines

A collection of 20 different wines, whites and reds from different grape varieties, were sampled in wineries (vats, barrels, and bottled final wines). Serial dilutions were subjected to Cells-qLAMP reactions as described above.

## Results

### Design and Specificity of the LAMP Primer Sets for *O. oeni*

Based on the 16S rRNA region and using the LAMP Designer 1.13 software, six LAMP primers (two outer, two inner, and two loop) were carefully designed (**Table 2**) for *O. oeni* specific detection purposes.

**Table 2:** Sequences of the LAMP primer sets.

Primer	Sequence (5′-3′)	Amplicon Size (bp)
Loo-F3	GATTTATTGGGCGTAAAGCG	307
Loo-B3	TGCTACGTCACTAGGAGG	
Loo-FIP	TTCACCGCTACACATGGAGTT CCTCGGAACTGCATTGGAA	212
Loo-BIP	GCGGCTTGCTAGATCGTAACTC AATCCCGTTTGCTACCC	
Loo-LoopF	GCCTCTATCGCACTCAAGTAA	124
Loo-LoopB	GACGTTGAGGCTCGAAAGTA	

*In silico* validation of the primers was carried out using BLAST tool against the EMBL/GenBank databases.

After the design, primers’ specificity was estimated by the LAMP reactions against the different species of bacteria and yeasts. Increased turbidity accompanied by DNA amplification was observed only when the primer set was reacted with the *O. oeni* species. No amplification was observed with the organisms other than the target species. The results were confirmed by resolving the amplified products by agarose gel electrophoresis which, as expected, displayed a ladder-like pattern only for *O. oeni* (data not shown).

### Direct *O. oeni* Detection

**FIGURE 1:**
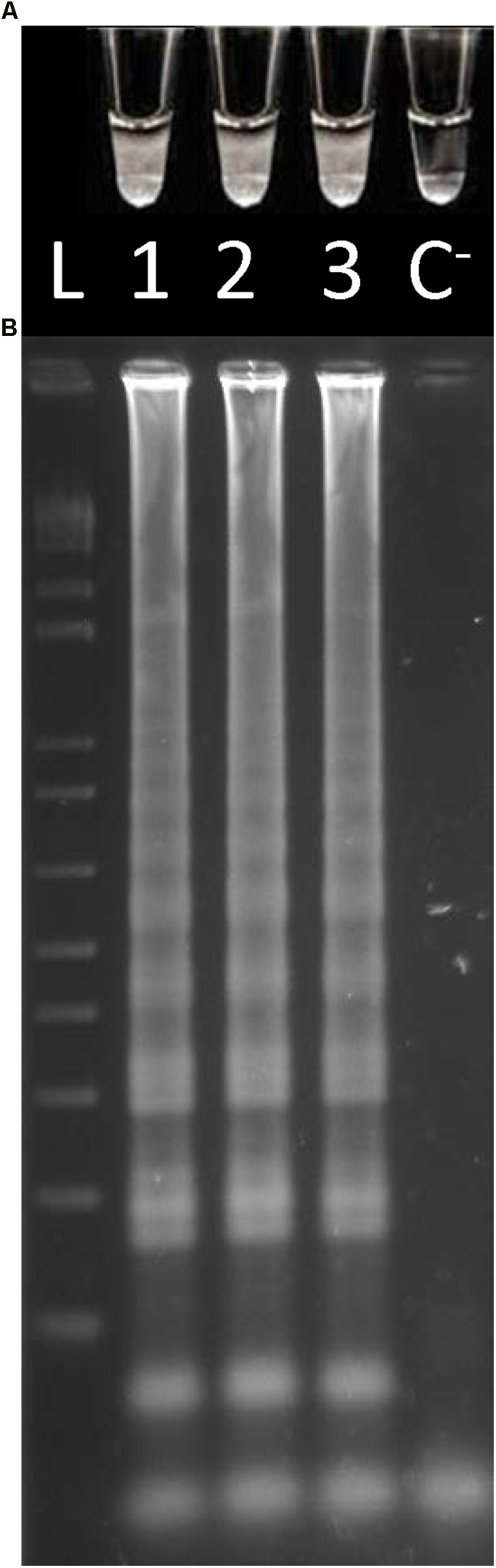
LAMP amplifications from the *O. oeni* cell suspensions from the culture medium and DNA. **(A)** Direct turbidity observation; **(B)** Agarose gel electrophoresis of the amplified products. 1, 2, culture medium; 3, DNA; 4, C^−^, negative control; L, 1 kb plus DNA Ladder (Invitrogen).

Considering that the designed primers were species-specific, the applicability of the Cells-LAMP method for the direct *O. oeni* total cells detection was verified by comparing the target products amplified from both cell suspensions (10^6^ cells/mL) and the DNA extracted from the same cell suspensions. For the extracted DNA, the sample volume used in each reaction was 2.5 μL as indicated by Hayashi et al. (2007). Nevertheless, regarding the whole cells, fivefold higher sample volumes were applied to each LAMP reaction to enhance the method’s sensitivity (Soares-Santos et al., 2018). For both DNA and cells, the results showed the presence of turbidity by directly observing the reaction tubes (**Figure 1**). This proved that it is possible to do away with the DNA extraction step for LAMP amplifications. Moreover, these results were confirmed when resolving the amplified products by agarose gel electrophoresis which, as expected, displayed a ladder-like pattern (**Figure 1**). The lack of both turbidity and a ladder-like pattern in the negative control sample confirmed the assay’s specificity.

**FIGURE 2:**
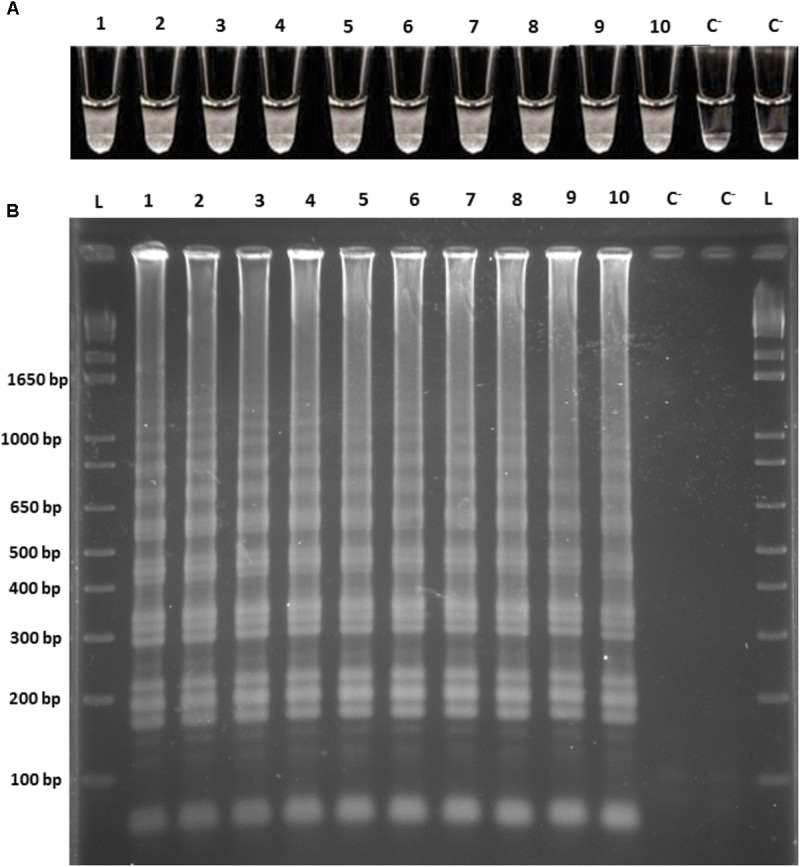
LAMP amplifications from the *O. oeni* cell suspensions from the culture medium, white grape must, red grape must, white wine, and red wine. **(A)** Direct turbidity observation; **(B)** Agarose gel electrophoresis of the amplified products. 1, 2, culture medium; 3, 4, white grape must; 5, 6, red grape must; 7, 8, white wine; 9, 10, red wine; C^−^, negative control; L, 1 kb plus DNA Ladder (Invitrogen).

Subsequently, the ability of the LAMP assay to directly detect *O. oeni* cells in the white/red grape musts and wines was evaluated. For this purpose, cell suspensions at a final concentration of 10^6^ cells/mL were prepared in all the different matrices in independent experiments. Two samples of the *O. oeni* cell suspensions from the culture medium were used as positive controls. The increased turbidity, along with DNA amplification, was observed in all the reaction tubes, along with the presence of a ladder-like pattern according to the agarose gel electrophoresis of the respective amplified products (**Figure 2**). Hence, we confirm the detection of the *O. oeni* whole cells by LAMP reaction in 1 h and directly from the wine-related matrices that overcomes the presence of the inhibitors inherent to such matrices.

### Limit of Detection of the *O. oeni* From the White and Red Wines

**FIGURE 3:**
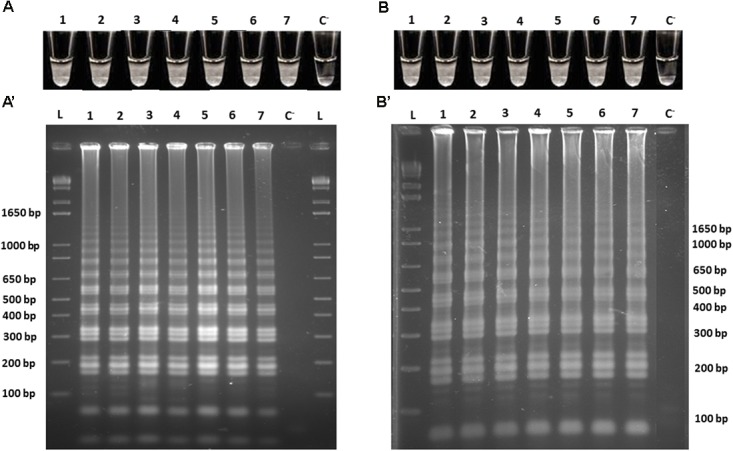
Cells-LAMP amplifications from the 10-fold serial dilutions of the *O. oeni* lysed cells in **(A)** white wine, and **(B)** red wine. A, B, direct turbidity observation; A′, B′, agarose gel electrophoresis of the amplified products; 1, 10^8^ cells/mL; 2, 10^7^ cells/mL; 3, 10^6^ cells/mL; 4, 10^5^ cells/mL; 5, 10^4^ cells/mL; 6, 10^3^ cells/mL; 7, 10^2^ cells/mL, C^−^, negative control; L, 1 kb plus DNA Ladder (Invitrogen).

In order to assess the limit of detection (LoD) of the Cells-LAMP method for detecting *O. oeni* in white and red wines, serial dilutions were prepared in both matrices and the same matrix was used as the diluent. After washing cells, all the cell suspensions were subjected to LAMP amplification with specific primers in independent experiments. The results were obtained by direct turbidity observation in the reaction tube, followed by product resolving by agarose gel electrophoresis. The obtained LoDs were 10^3^ cells/mL (12 cells/reaction tube) in white wine, and 10^4^ cells/mL (125 cells/reaction tube) in red wine (data not shown). To lower the *O. oeni* LoD in both matrices, the effect of a previous cell wall mechanical lysis was evaluated (Soares-Santos et al., 2018). To this end, the assay was repeated, but before the Cells-LAMP amplification, all the cell suspensions were subjected to a mechanical lysis. In both experiments, the obtained results showed that the cell lysis lowered the *O. oeni* LoD for 10^2^ cells/mL in both matrices (1 cell/reaction tube). Therefore, the LoDs were 10- and 100-fold lower in the white and the red wine, respectively (**Figure 3**), and showed excellent assay sensitivity when a mechanical lysis was performed.

### Direct *O. oeni* Quantification

By considering the possibility of detecting *O. oeni* cells in the wine-related matrices by LAMP, the availability of the Cells-qLAMP assay for the direct quantification of this species was also investigated. For this purpose, the cell suspensions at a final concentration of 10^3^, 10^5^, and 10^7^ cells/mL were prepared in culture media, washed, mechanically lysed, and then subjected to qLAMP amplification using SYBR Green I or SYTO-9 as fluorescent dyes in independent experiments.

**FIGURE 4:**
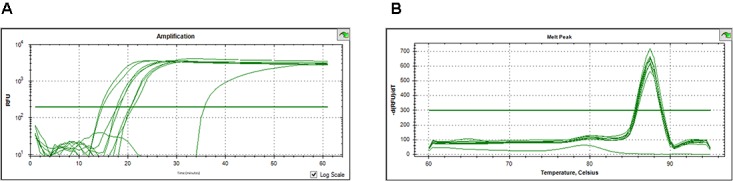
Results of the *O. oeni* detection and quantification by SYTO-9 fluorescence-based Cells-qLAMP. **(A)** Relative fluorescence units (RFU) vs. time; **(B)** The melting curves analysis of the amplified products.

The obtained results showed that the qLAMP assay run with SYBR Green I could not efficiently detect and quantify *O. oeni* cells. Moreover, the melt peaks also indicated lack of specific amplification (data not shown). Nevertheless, the amplification curves obtained for the SYTO-9 qLAMP reactions indicated positive results as typical real-time amplification performance was observed (**Figure 4A**), and the observed melt peaks were also specific (**Figure 4B**). A good correlation was observed between the Tt of the qLAMP reaction and the *O. oeni* cells’ concentration, which suggests that amplification was reliable and the SYTO-9 fluorescence-based qLAMP assay could be used for the detection and quantification of *O. oeni* cells.

**FIGURE 5:**
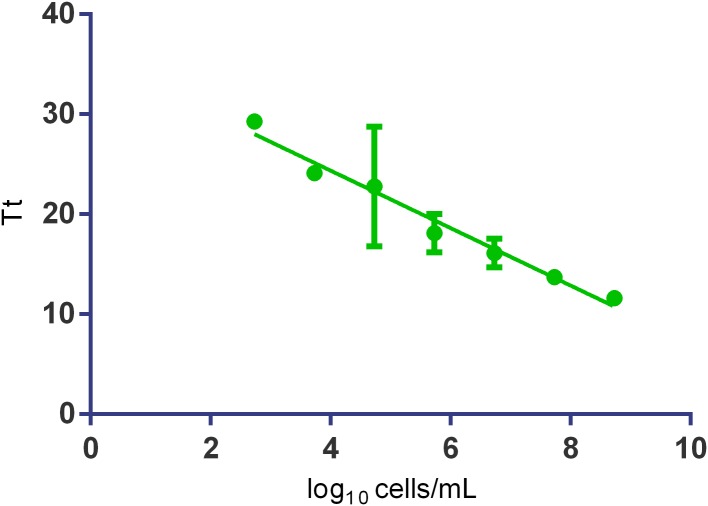
The standard curves obtained by SYTO-9 fluorescence-based Cells-qLAMP from the 10-fold serial dilutions of *O. oeni* in the culture medium. The Tt values are the averages of three replicates. Error bars represent standard errors.

Afterward the sensitivity of the SYTO-9 fluorescence-based qLAMP to detect and quantify *O. oeni* cells was evaluated. For the analysis, the 10-fold serial diluted cells from 10^8^ to 10^2^ cells/mL were prepared in the culture medium, washed, mechanically lysed, and subjected to qLAMP amplification. The data regression analysis (**Figure 5**) showed that the assay was linear over seven orders of magnitude (10^2^ to 10^8^ cells/mL or 1 to 10^6^ cells/reaction) with an *R*^2^ over 0.97 and an *E* of 1.23.

**FIGURE 6:**
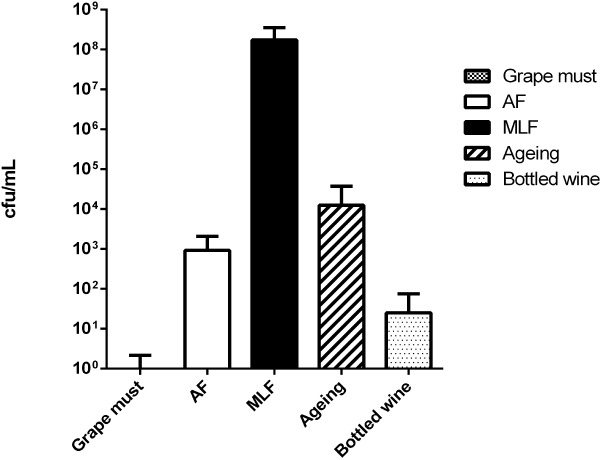
Results of the *O. oeni* quantification by Cells-qLAMP of different grape musts and wines, both whites and reds, sampled at different winemaking stages. (Grape must) initial phases; (AF) during alcoholic fermentation; (MLF) during malolactic fermentation; (aging) during the aging in barrels; (bottled wine) final wines already bottled.

Real wine samples were then analyzed with Cells-qLAMP to detect and quantify *O. oeni* cells (**Figure 6**). In all the samples where MLF was in progress, these cells were easily detected and quantified. In early winemaking stages (i.e., grape must and early alcoholic fermentation) and very late aging or bottled old wines, not always *O. oeni* cells were detected and quantified, even when concentrating samples 10× or 100×.

## Discussion

Although PCR-based methods are widely used, they require a thermocycler to carry out DNA amplification via specific temperature phases and, in some cases, the result needs to be further analyzed by electrophoresis, plus image capturing and analysis. LAMP is a promising technique for the accurate detection and quantification of microorganisms that requires only four primers, a DNA polymerase, and a regular laboratory water bath or heat block for reactions. Moreover, the LAMP reaction combines specificity, sensitivity, and efficiency (Parida et al., 2008; Notomi et al., 2015; Kumar et al., 2017), and can be implemented even after eliminating DNA extraction, which considerably cuts the overall assay time and reaction cost (Poon et al., 2006; Hill et al., 2008; Dugan et al., 2012). Nevertheless, wine is a complex matrix that contains DNA amplification inhibitors (Wilson, 1997) and, to date, LAMP application from wine samples requires some sort of nucleic acid extraction before starting reactions (Hayashi et al., 2007). In this study, a LAMP method for the direct and rapid detection and quantification of *O. oeni* was developed for the first time and was successfully applied to avoid DNA extraction steps.

The six designed LAMP primers to target the 16S rRNA gene were highly specific for *O. oeni* as no amplification with DNA from the other 10 bacteria and yeast species was obtained, whereas the DNA from *O. oeni* was efficiently amplified. The LAMP method developed with this primer set allowed the direct detection of *O. oeni* cells as turbidity and a ladder-like pattern were observed for both DNA and cells. These results confirmed that it is possible to do away with the DNA extraction step for LAMP amplifications. The results of those LAMP amplifications done directly from white/red grape musts and wines also confirmed the method’s reliability and validity to detect *O. oeni* whole cells directly from wine-related matrices. Nevertheless, when the LoDs of the LAMP method for *O. oeni* detection in white and red wines were assessed, differences in assay sensitivity were observed. For the white wine, the LoD was 10^3^ cells/mL (12 cells/reaction tube) and was 10^4^ cells /mL (125 cells/reaction tube) for red wine. This result may be due to the PCR inhibitors present in wine-related matrices, such as tannins, polysaccharides, polyphenols and ethanol (Wilson, 1997; Demeke and Jenkins, 2010; Schrader et al., 2012), and to the fact that their inhibitory effect is stronger in red wines with a higher concentration of polyphenols that in white wines. An increase in LAMP assay sensitivity was achieved in both matrices by a previous mechanical cells wall disruption as the LoD lowered by 10^2^ cells/mL. The LoD was higher than that obtained by Hayashi et al. (2007), who detected DNA in wine samples with 10 CFU/mL of *B. bruxellensis*. Nevertheless, it is important to consider that 10^2^ cells/mL means that it is possible to detect as few as 1-2 cells per reaction tube, which implies excellent assay sensitivity when performing a mechanical lysis with no DNA purification step. A concentration can be obtained in the centrifugation steps before running the LAMP reaction when resuspending cells in smaller volumes than the original ones. In short, the obtained results showed that the developed Cells-LAMP method allowed the direct detection of *O. oeni* cells from both grape musts and wines within 1 h from the start. The results of observing turbidity at the reaction endpoint were in accordance with that obtained by agarose gel electrophoresis. These results show that turbidity observation is a reliable method to visualize LAMP amplifications, and confirmation of the results by agarose gel electrophoresis can be ruled out for detection purposes. Moreover, agarose gel electrophoresis requires an expensive laboratory infrastructure (electrophoresis equipment, image capturing system, etc.), and generally toxic agents (ethidium bromide), which make the procedure impracticable for applications in low-resource wineries. Nevertheless, it is important to bear in mind that agarose gel electrophoresis can be useful for specificity evaluations as it can distinguish between real and false-positives in some unexpected cases when non-specific amplification occurs (Zhang et al., 2014).

Regarding *O. oeni* quantification, better results were obtained by the SYTO-9 fluorescence-based qLAMP than with SYBR Green I. These results agree with those obtained by Oscorbin et al. (2016) because the comparative study of six fluorescent dyes for qLAMP (SYTO-9, SYTO-13, SYTO-82, SYBR Green I, SYBR Gold, and EvaGreen), SYTO-9 and SYTO-82 gave the best results.

The standard curve constructed to evaluate the method’s sensitivity showed that the assay was linear over seven orders of magnitude (10^2^ to 10^8^ cells/mL). This result highlights the method’s high sensitivity as it indicates the possibility of detecting one cell per reaction. The Cells-qLAMP was also applied to real grape musts and wines, both whites and reds, with no inhibition from wine components. In some samples with low cell concentrations, it was possible to concentrate the cells, and then a quantification was possible.

## Conclusion

The designed primers allow the specific detection of *O. oeni* cells. The developed LAMP method allows *O. oeni* to be detected directly from both grape musts and wines within 1 h from the time the LAMP reaction begins, requires no DNA extraction and purification requirements. Nevertheless, the LAMP methodology’s high sensitivity was obtained by a previous mechanical cells lysis. The method was able to detect one single cell per reaction in culture media, and with white/red grape musts and wines with no reaction inhibition by ethanol, polyphenols, and other wine inhibitors. Nor does the LAMP method require expensive equipment and it can be easily operated. The developed SYTO-9 fluorescence-based qLAMP method offers the reliable direct detection and quantification of even 1 cell/reaction tube of *O. oeni*. Cells may be concentrated before the LAMP reaction to further increase this LoD. The developed method is both economic and fast, with high sensitivity and specificity. It can be transferred to serve companies in the wine sector because it allows results to be obtained during short periods so that the people responsible for production can make decisions on time.

## Author Contributions

All authors contributed to conception and design of the study. VS-S performed the statistical analysis and wrote the first draft of the manuscript. All authors contributed to manuscript revision, read, and approved the submitted version.

## Conflict of Interest Statement

The authors declare that the research was conducted in the absence of any commercial or financial relationships that could be construed as a potential conflict of interest.
